# Molecular Epidemiology of *Giardia, Blastocystis* and *Cryptosporidium* among Indigenous Children from the Colombian Amazon Basin

**DOI:** 10.3389/fmicb.2017.00248

**Published:** 2017-02-21

**Authors:** Angie Sánchez, Marina Munoz, Natalia Gómez, Juan Tabares, Laura Segura, Ángela Salazar, Cristian Restrepo, Miguel Ruíz, Patricia Reyes, Yuchen Qian, Lihua Xiao, Myriam C. López, Juan D. Ramírez

**Affiliations:** ^1^Facultad de Medicina, Departamento de Salud Pública, Universidad Nacional de ColombiaBogotá, Colombia; ^2^Grupo de Investigaciones Microbiológicas-UR, Programa de Biología, Facultad de Ciencias Naturales y Matemáticas, Universidad del RosarioBogotá, Colombia; ^3^Division of Foodborne, Waterborne, and Environmental Diseases, National Center for Emerging and Zoonotic Infectious Diseases, Centers for Disease Control and PreventionAtlanta, GA, USA

**Keywords:** *Giardia*, *Blastocystis*, *Cryptosporidium*, molecular epidemiology, assemblages, subtypes

## Abstract

The incidence and prevalence of intestinal parasites in children is most likely due to lack of natural or acquired resistance and differences in behavior and habits closely related to environmental and socioeconomic determinants. The most important protozoa that parasitize humans are *Giardia, Entamoeba, Blastocystis*, and *Cryptosporidium*. These parasites present wide intraspecific genetic diversity and subsequently classified into assemblages and subtypes. The Amazon basin is the largest in the world and is the fifth freshwater reserve on the planet. Contradictorily, people living in these areas (Indigenous populations) have poor quality of life, which favors the infection of diseases of fecal-oral transmission. The aim of this work was to unravel the molecular epidemiology of *Giardia, Blastocystis* and *Cryptosporidium* across four communities (Puerto Nariño, San Juan del Soco, Villa Andrea and Nuevo Paraíso). We obtained 284 fecal samples from children under 15 years old that were analyzed by direct microscopy (261 samples) and Real Time PCR (qPCR) (284 samples). The positive samples for these protozoa were further characterized by several molecular markers to depict assemblages and subtypes. We observed a frequency of *Giardia* infection by microscopy of 23.7% (62 samples) and by qPCR of 64.8% (184 samples); for *Blastocystis* by microscopy of 35.2% (92 samples) and by qPCR of 88.7% (252 samples) and for *Cryptosporidium* only 1.9% (5 samples) were positive by microscopy and qPCR 1.8% (5 samples). Regarding the *Giardia* assemblages, using the glutamate dehydrogenase (*gdh)* marker we observed AI, BIII and BIV assemblages and when using triose phosphate isomerase (*tpi*) we observed assemblages AI, AII, BIII and BIV. In contrast, *Blastocystis* STs detected were 1, 2, 3, 4, and 6. Lastly, the species *C. viatorum, C. hominis* (with the subtypes IdA19 and IaA12R8) and *C. parvum* (with the subtype IIcA5G3c) were identified. We observed a high profile of zoonotic transmission regarding the *Giardia* assemblages and *Blastocystis* STs/alleles. Also, we highlight the elevated frequency of infection by these two protozoans suggesting an active transmission in the area. Our findings reinforces the need to deploy better epidemiological surveillance systems for enteric pathogens in the area.

## Introduction

Over 15 genera of protozoa parasitizing humans are known, some of these are considered natural commensal and others are related to intestinal infections responsible for generating symptoms in the infected hosts. The most frequent protozoans with cosmopolitan distribution are *Giardia, Entamoeba*, and *Blastocystis*. In Colombia, as reported in the latest national survey of intestinal parasitism in school population for the years 2012–2014, the most prevalent intestinal protozoan pathogen was *Blastocystis* with 57.7%, the second was *Entamoeba histolytica/dispar/moshkovskii* complex (*Entamoeba* complex) with 17% and the third was *Giardia* with a national prevalence of 15.4% (Ministerio de Salud y Protección Social, 2015). Additionally, *Cryptosporidium* has been reported with a frequency of 0.5% in the country. Currently, these are considered emerging opportunistic parasites of importance in public health at international level (Yoder et al., 2012). Factors associated with these parasitic infections are usually fecal contamination of soil and food, access to drinking water, wastewater use, lack of sanitation and vulnerable socio-economic conditions. Thus, maintaining the prevalence of intestinal parasitic infections in populations with these characteristics generating a latent risk to endure a dynamic transmission among its inhabitants (Ortiz et al., 2012).

One of the most common parasitic diseases in different geographical areas worldwide is giardiasis. It is estimated that 200 million people have symptoms and approximately 500,000 new cases per year are reported (Casero et al., 2015). In a retrospective study sought to describe the incidence of giardiasis in Colombia, for the years 2009–2013 revealed a report of 15,851 cases, of these, 50.3% were women; 58.4% were under 10 years old and 14.8% were 10–19 years old. Some survey data indicate that in industrialized countries, the prevalence ranges between 2 and 5% and the rate for developing countries ranges from 20 to 30% (Rodríguez-Morales et al., 2016). This disease is caused by *Giardia intestinalis*, an enteric pathogen that parasitize humans, domestic animals and wildlife. Studies in *Giardia* isolates have allowed to characterize the genetic diversity of the parasite, based on molecular markers such as the small subunit rRNA, glutamate dehydrogenase (*gdh*), triose phosphate isomerase (*tpi*) and β-giardin (Ryan and Caccio, 2013), allowing to identify eight assemblages where A and B are related to infections in humans and animals and further subdivided into sub-assemblages: AI, AII, BIII, and BIV with no strict associations based on clinical presentation (Mayrhofer et al., 1995; Thompson, 2000). Assemblages C and D infecting dogs, assemblage E infecting ruminants and pigs; Assemblages F infecting cats; G infecting rodents and assemblage H infecting seals and gulls (Monis et al., 2009). These parasitic subpopulations are of epidemiological significance as a potential zoonosis and wide distribution in domestic animals that generate different transmission routes (Ramírez et al., 2015). This re-emerging parasite is a protozoan that produces symptomatic conditions such as diarrhea, abdominal pain and malabsorption syndrome (Stensvold, 2013; Roberts et al., 2014).

Likewise, the blastocystosis is an intestinal colonization caused by a stramenopile known as *Blastocystis* which is often observed in fecal samples from humans and animals; with a worldwide distribution frequency reaching 100% in developing countries and exceeding 56% in developed countries (Scanlan et al., 2014). *Blastocystis* pathogenicity remains controversial, some authors suggest that the presence of infection might cause diarrhea, flatulence, bloating, urticaria and irritable bowel syndrome (IBS) (Casero et al., 2015). However, recent microbiome studies suggest that *Blastocystis* colonization is usually associated with a healthy gut microbiota, rather than with gut dysbiosis generally observed in metabolic or infectious inflammatory diseases of the lower gastrointestinal tract (Audebert et al., 2016). Also, a metagenomics approach showed that individuals with intestinal microbiota dominated by *Bacteroides* were much less prone to having *Blastocystis*-positive stool than individuals with *Ruminococcus* and *Prevotella*-driven enterotypes showing that the presence of *Blastocystis* might be benefitial for the human health (Andersen et al., 2015). *Blastocystis* has been divided into subtypes (STs) based on 18S rDNA polymorphisms and so far, 17 STs have been identified. The STs related to infections in humans and animals are 1-9 and 12, and the STs 10, 11, 13–17 have only been detected in animals (Scanlan et al., 2014). However, the pathogenicity of this organism is under strong debate, mainly due to a high rate of asymptomatic carriers, the differences in host susceptibility, intestinal microbiota and/or different pathogenic potential of different genetic STs (Stensvold et al., 2012; Ramírez et al., 2014). In the case of Colombia, the reported studies suggest the existance of STs 1, 2, 3, 4, 6, and 7 (Ramírez et al., 2016).

Lastly, *Cryptosporidium* is a cause of cryptosporidiosis. A diarrheal disease affecting especially in children under 5 years and patients with immune deficiencies (Kotloff et al., 2013). In the developed countries, diarrhea is the most common reason for missing work, while in the developing world, it is a leading cause of death. Internationally, the mortality rate is 5–10 million deaths each year (Nemes, 2009). In this scenario, *Cryptosporidium* is a major cause of diarrheal disease, globally (Shirley et al., 2012). Unlike many common causes of infectious enteritis, control and treatment of this infection are still problematic. Different reports indicated that cryptosporidiosis causes more than 99,000 deaths and 8.3 million disabilities (adjusted by years of life) by 2010; Most of these deaths and disabilities were common in developing countries (Ghenghesh et al., 2016). The course of infection may be intra and extra intestinal, symptomatic and asymptomatic in some individuals causing secretory diarrhea and malabsorption syndrome (Davies and Chalmers, 2009).

Molecular tools have been used to discriminate and subtype *Cryptosporidium* species. One of the popular subtyping tools is the DNA sequence analysis of the 60 kDa glycoprotein (gp60, also called gp40/15); Gp60 is found on the surface of the apical region of invasive phases of the parasite, and one of the dominant targets for neutralizing antibody responses in humans (Wanyiri et al., 2007). Currently, nearly 20 *Cryptosporidium* species and genotypes have been reported in humans, including *C. hominis, C. parvum, C. meleagridis, C. felis, C. canis, C. cuniculus, C. ubiquitum, C. viatorum, C. muris, C. suis, C. fayeri, C. andersoni, C. bovis, C. scrofarum, C. tyzzeri, C. erinacei*, and *Cryptosporidium* I genotypes from horse, skunk, and chipmunk (Xiao, 2010; Liu et al., 2014; Ryan and Hijjawi, 2015). Within each subtype family, subtypes differ from each other mostly in the number of trinucleotide repeats (TCA, TCG, or TCT microsatellite; Xiao, 2010).

The incidence and prevalence of intestinal parasites in children is most likely due to lack of natural or acquired resistance and differences in behavior and habits closely related to environmental and socioeconomic determinants. In Colombia, there is information of the most common intestinal parasites but still lacks information about parasitic subpopulations (genotypes, subtypes, or assemblages) circulating in the population. In the case of the Colombian Amazon, it is not known in indigenous communities the frequency of different intestinal parasites that may be present in these ethnic groups, which are at risk of infection because of living in inappropriate conditions, associated with fecal contamination of soil and food, inadequate drinking water, wastewater use, lack of sanitation and vulnerable socioeconomic conditions. Thus, the prevalence of intestinal parasitic infections in populations with these sociodemographic characteristics is maintained, creating a risk for transmission dynamics among its inhabitants and a public health problem in the country (Rinne et al., 2005). Therefore, the aim of this study was to perform molecular diagnosis and genotyping from stool samples positive for *Giardia, Blastocystis*, and *Cryptosporidium* in children under 15 years of four indigenous communities in the Colombian Amazon.

## Materials and methods

### Study population

**Figure 1:**
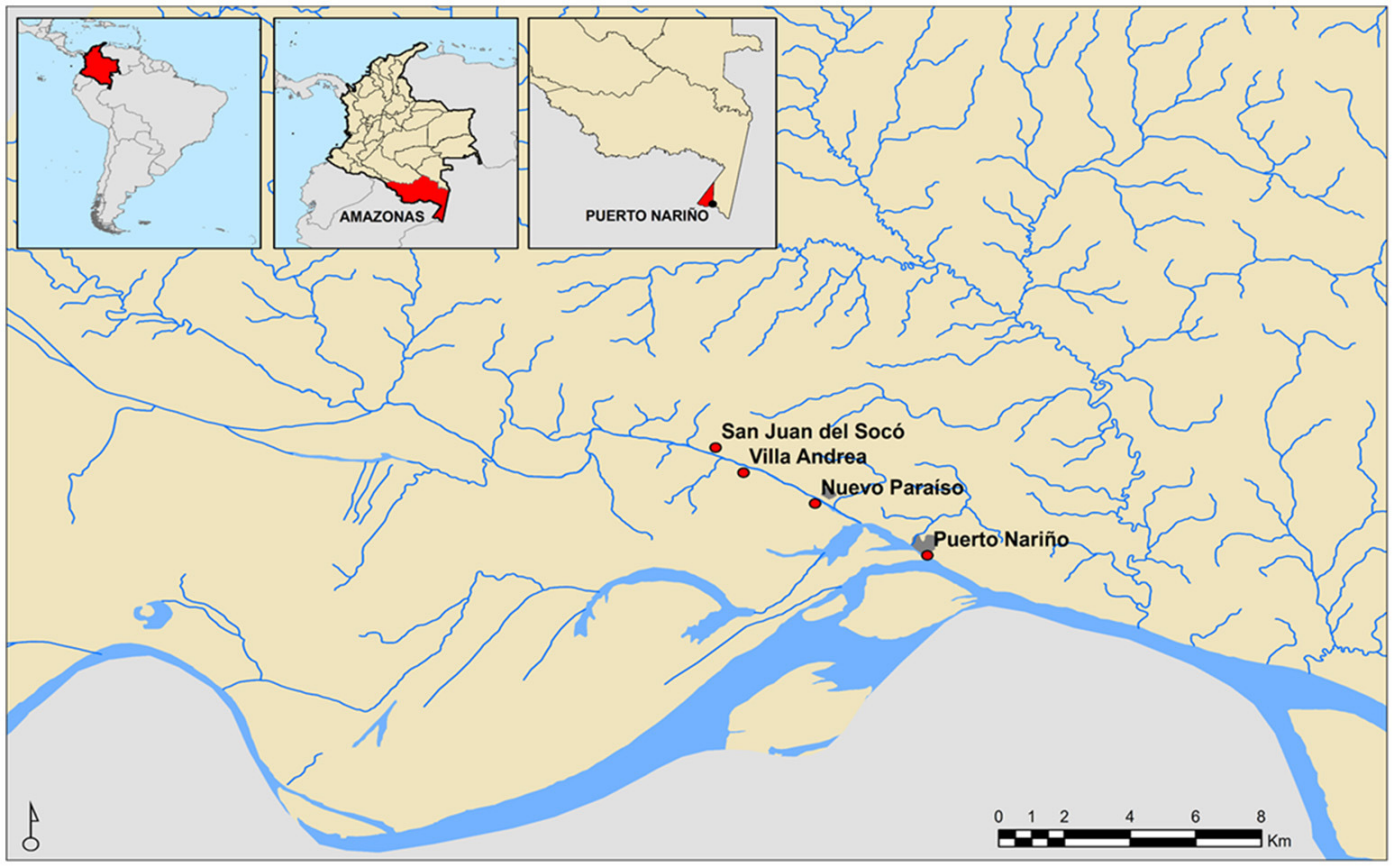
**Geographical location of Indigenous communities participating in the study**.

Convenience sampling was conducted due to the geographical proximity of the individuals (Amazonas, Colombia) and age group (under 15 years). Therefore, we randomly selected 284 stool samples. The age average of the population included was 7.3 years (SD: 4.2 years—range 1–15). Individuals included in the study belonged to the municipality of Puerto Nariño located in the department of Amazonas, in three rural settlements linked to the jurisdiction of the Association of Indigenous Authorities of Tikuna, Cocama, and Yagua. According to the sampled communities the following distribution in sample number was obtained for every rural settlement: Nuevo Paraíso 10.9% (*n* = 31), Villa Andrea 15.1% (*n* = 43), San Juan del Socó 28,2% (*n* = 80) and the municipal capital Puerto Nariño 45.8% (*n* = 130), which are located around the river Loretoyacu which has its confluence with the Amazon River (Figure 1).

### Ethical considerations

This study is an investigation of minimal risk to participants, in which the ethical standards set by the Ministry of Health of Colombia, the Juvenile Code and the Declaration of Helsinki of 2013 were followed. The parents/guardians of the minors provided informed consent and assent in order to obtain the sample. This project has approval certificate from the ethics committee of the Universidad Nacional de Colombia under the number: 002-012-15 issued on February 12, 2015.

### Collection, conservation, and DNA extraction of the samples

The samples were stored and labeled in plastic containers, transported to the laboratory of Parasitology of the Universidad Nacional de Colombia for further processing. They were divided into two parts, one part of the sample was used for diagnosis by microscopy; The second part was fixed in proportion weight–volume (1:4 stool:alcohol 70%) and were stored at −20°C for DNA extraction from 300 μL of stool by using the kit NORGEN Stool DNA Isolation, following the manufacturer's instructions.

### Diagnosis by microscopy and real-time PCR for identification of *Giardia, Blastocystis*, and *Cryptosporidium*

The conventional routine microscopic diagnosis was conducted across 261 of 284 samples collected by direct examination in saline solution and lugol for identifying protozoa, and Ziehl Neelsen for identifying *Cryptosporidium* oocysts. Real time PCR was performed in all the 284 samples collected for *Giardia, Blastocystis* and *Cryptosporidium* by TaqMan system using previously reported primers and probes (Mejia et al., 2013). The qPCRs were performed in 96 wells MicroAmp (Applied Biosystems), reactions in a total volume of 9 μL containing 3.5 μL of Taqman™ Mastermix (Roche), 1.0 μL of species-specific primers (10 μM) and primers of the internal amplification control (IAC) (10 μM), and 0.4 μL Taqman probes (5 μM) (*Giardia, Blastocystis, Cryptosporidium* and the IAC), 0.3 μL the water and 2.0 μL of DNA. The samples were processed by duplicate in an Applied Biosystems 7,500 Fast equipment using default parameters of 40 cycles. The qPCR results were considered negative if the cycle threshold values (Ct) were >38 (Mejia et al., 2013). To corroborate the Ct value, we conducted experiments to establish the dynamic range of our assay using standards from 10,000 to 1 fg/μL. For quantification, plasmids containing the target sequences were cloned into the pGEM–T Easy Vector System I (Promega, UK), according to the manufacturer's instructions, and transformed into XL1-Blue Escherichia coli (Agilent Technologies, UK). The transformed colonies containing the plasmids were extracted by using the QIAprep Spin Miniprep Kit (QIAGEN, Valencia, CA). The purified plasmid DNA was quantified by using a Nanodrop and diluted to have a concentration range of 10,000 to 1 fg/μL. The dynamic range established that the limit of detection was the proposed by Mejia et al. (2013). We employed as positive controls DNA from *Giardia, Blastocystis* and *Cryptosporidium*; and as negative controls fecal negative samples of patients from non-endemic regions that were previously tested by microscopy and qPCR.

### Genotyping for identifying *Giardia* assemblages, *Blastocystis* STs and alleles, and *Cryptosporidium* species and subtypes

As inclusion criterion, the genotyping was conducted in those positive samples obtained by real-time PCR for *Giardia, Blastocystis*, and *Cryptosporidium*. Any positive sample was subjected to conventional PCR to obtain amplification products using primers of the following molecular markers: *gdh* (Glutamate dehydrogenase) using primers GDHeF (5′-TCAACGTYAAYCGYGGYTTCCGT-3′), GDHiF (5′-CAGTACAACTCYGCTCTCGG-3′) and GDHiR (5′-GTTRTCCTTGCACATCTCC-3′) as reported elsewhere (Feng and Xiao, 2011) and *tpi* (triose phosphate Isomerase) using primers AL3543 (5′-AAATIATGCCTGCTCGTCG-3′), AL3546 (5′-CAAACCTTITCCGCAAACC-3′), AL3544 (5′-CCCTTCATCGGIGGTAACTT-3′), AL3545 (5′-GTGGCCACCACICCCGTGCC-3′) as reported elsewhere (Feng and Xiao, 2011) for the identification of *Giardia* assemblages. In the case of *Blastocystis*, SSU rRNA amplification was performed with primers RD5 (5′-ATCTGGTTGATCCTGTCCAG-3′) and BhRDr (5′-GAGTGCCTTTTT AACAACAAC G-3′) for identifying the STs and alleles of *Blastocystis* as previously recommended (Scicluna et al., 2006). In the case of *Cryptosporidium*, species were identified by using a small subunit rRNA-based PCR–restriction fragment length polymorphism genotyping tool (Chalmers and Katzer, 2013). Subtyping of *C. hominis* and *C. parvum* was based on sequence analysis of GP60 genes. Each specimen was analyzed by either method at least twice. Subtype families within *C. hominis* and *C. parvum* were determined on the basis of sequence differences in the nonrepeat region of the gene. Within each subtype family, subtypes differed from each other, mostly in the number of serine-coding trinucleotide repeats (TCA, TCG, or TCT microsatellite) located in the 5′ region of the gene. The previously established nomenclature system was used to differentiate subtypes within each subtype family (Xiao, 2010).

The PCR products were sequenced using the dideoxy-terminal method in a 310 Genetic Analyzer (Applied Biosystems) using both strands, the sequences obtained were edited and aligned in MEGA 5.0 (Tamura et al., 2011) and compared with reference sequences by BLAST. In addition, for *Blastocystis*, queries were performed on the database available for obtaining alleles and STs confirmation (http://pubmlst.org/blastocystis/). Phylogenetic reconstruction was performed using Maximum Likelihood with 1,000 bootstrap replicates with reference sequences contained in GenBank with the following accession numbers: AI (M84604), AII (AY178737), BIII (AF069059), BIV (AY178739), C (U60982), D (U60986), E (AY178741), E (AB182127), F (AB569384), G (AF069058), G (AY178745), H (GU176089) and rooted with *G. microti* (AY228649.1) and *G. ardeae* (AF069060). In the case of *Cryptosporidium*, sequences were compared with species and subtype control sequences harbored at CDC, Atlanta. The sequences were deposited under the accession numbers KX963577–KX963768.

### Genetic diversity indexes in *Giardia* and *Blastocystis*

We calculated the genetic diversity indexes per community across the *gdh* and *tpi* markers for *Giardia* and 18S rDNA marker for *Blastocystis*. π and θ nucleotide diversity indexes and haplotype diversity were calculated in DNAsp v.5.0.

### Statistical analysis

Descriptive statistics was used to describe the main events of interest. Categorical variables are reported in terms of percentages, with corresponding Confidence Intervals (CI) at 95%, calculated using the bootstrap method. In the case of continuous variables, means were calculated with their corresponding measures of dispersion (standard deviation-SD). The existence of association between categorical variables were evaluated using chi-square or Fisher, as appropriate tests. The distribution obtained by frequency for females was 49.2% (*n* = 128) and 50.8% (*n* = 132) for male. The existence of association between the main results (epidemiological data and infection results) was evaluated using the categorical variables using chi-square or Fisher, tests, according to the characteristics of the data. In the case of the age of the individuals, was necessary to categorize the variable considering distribution percentiles obtaining four groups of 1–4 years, 5–8 years, from 9 to 12 years and one finally greater than or equal to 13 years old. First, the frequency of infection by age group, sex and community was obtained. Once identified the frequency and distribution of infection in each community were conducted bivariate analyzes to determine the existence of association between parasitic infections and socio-demographic factors (age, sex and community) for infected individuals. The associations between the results of infection (including the genetic diversity indexes (tpi, gdh, and 18S) within the groups of the same sex or age was identified per community. We employed chi-square or Fisher tests. In order to assess the presence and distribution of assemblages of *Giardia* and STs of *Blastocystis*, descriptive analysis were performed and association tests were developed according with the test previously mentioned, with the aim to compare against each demographic factor, likewise proceeded for STs present in *Blastocystis*. In the case of finding any degree of significance with STs, alleles belonging to it were faced with each variable in order to find some degree of association between a particular allele in populations. Lastly, we compared the presence of intestinal parasites such as *Ascaris, Trichuris*, hookworms and *Entamoeba* complex with *Giardia* assemblages, *Blastocystis* STs and *Cryptosporidium* species. The difference in the genetic diversity indexes was evaluated through comparison tests between independent samples, considering the results for each community. A value of *p* < 0.05 was considered statistically significant for all hypothesis testing. Statistical analysis was performed using STATA version 10.1 (Stata Corp, College Station, TX).

## Results

### Frequency of intestinal parasites in samples

A total of 261 samples were analyzed by microscopy over the 284 collected because not enough sample was obtained for the direct analysis. In general, for stool samples collected in the school population of the four indigenous communities there were identified by microscopy other intestinal parasites such as: Whipworm in 62.0% (*n* = 162), *Ascaris lumbricoides* in 46.3% (*n* = 121), hookworms in 22.2% (*n* = 58) and *Entamoeba* complex in 20.7% (*n* = 54). At the time of the analysis in order to find any kind of association between the intestinal parasites (*Trichuris, Ascaris*, hookworms and *Entamoeba* complex) and the *Giardia* assemblages, *Blastocystis* subtypes and *Cryptosporidium* species, no significant association was found between the frequencies of the other parasites reported by microscopy.

### Frequency of *Giardia* infection

**Figure 2:**
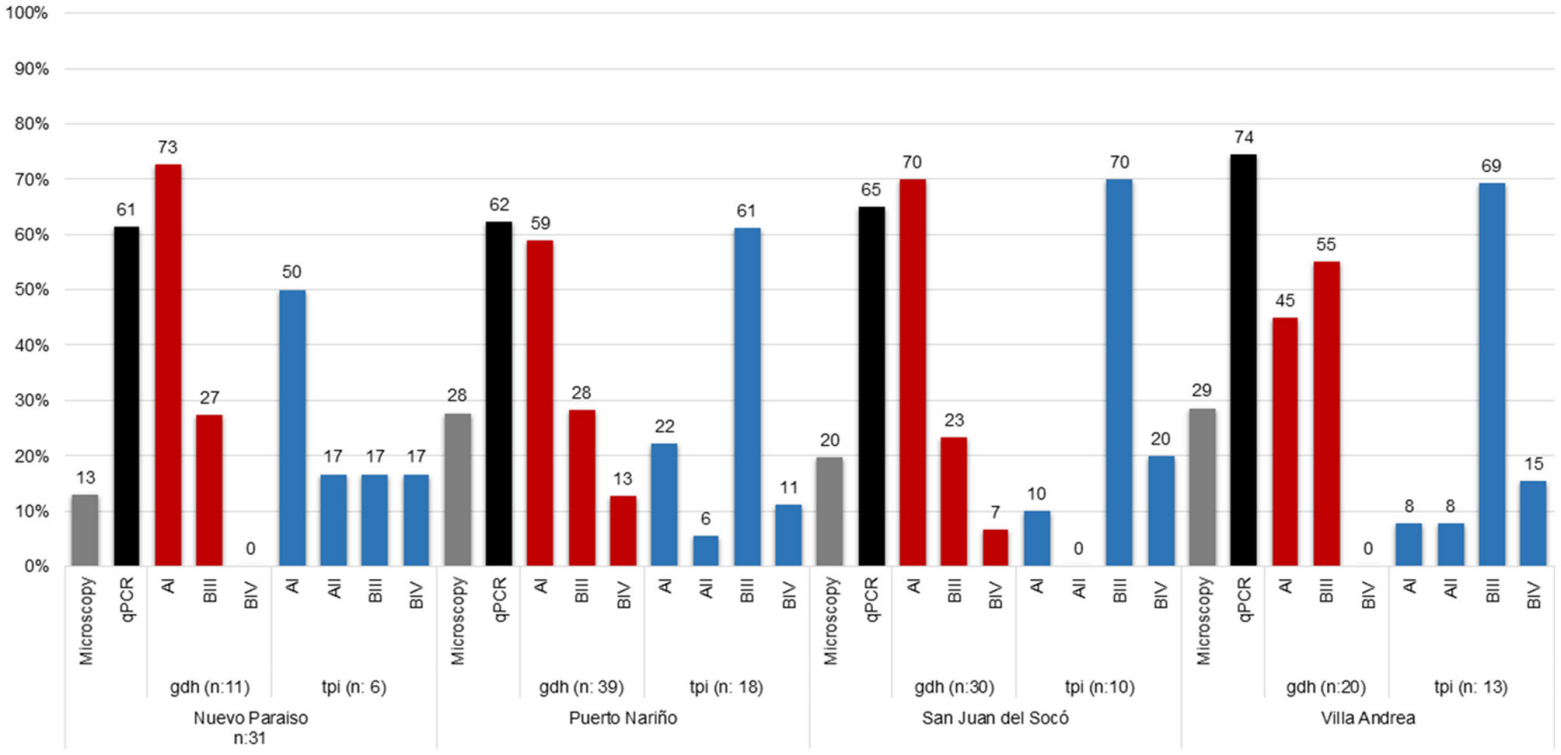
**Frequency and Distribution of ***Giardia*** assemblages for each of the studied communities**.

Sixty-two samples were microscopy positive for *Giardia* (23.7%, CI:18.7–29.4) demonstrating that the highest percentage of infection was in the community of Villa Andrea (28.6%) and Puerto Nariño (27.7%) followed by San Juan del Socó (19.7%) and Nuevo Paraíso (12.9%). For Real-Time PCR, 184 samples (64.8%, CI: 58.9–70.3) were positive, similarly observing the highest percentage of infection for Villa Andrea (74.4%) and San Juan del Socó (65.0%), followed by Puerto Nariño (62.3%) and Nuevo Paraíso (61.3%). The most common assemblage was AI (61%) (Table 1; Figure 2), more frequently present in the community of San Juan del Socó (40.4%), the second most common assemblage was BIII (17.2%) present more frequently in the community of Villa Andrea (34.4%). There was no significant association between the frequency of infection and communities. Across the positive samples, 120 sequences were analyzed with *gdh* finding the AI, BIII and BIV assemblages showing a significant association between communities (Table 2). The *tpi* marker showed the presence of the four assemblages AI, AII, BIII, and BIV for which the most frequent assemblage was BIII (59.6%) (Table 2), more frequently present in the community of Villa Andrea (28,1%) followed by AI (4.9%) more frequently present in the community of Nuevo Paraíso (16.7%). No significant association between assemblages and communities were found with the markers employed. Additionally, the degree of agreement between the two markers used for genotyping (gdh and tpi) based on a chi-square test showed no association between the two markers (*p* = 0.135).

Table 1**Frequency of infection by microscopy and qPCR of ***Giardia, Blastocystis*** and ***Cryptosporidium***; and ***Giardia*** assemblages, ***Blastocystis*** subtypes and ***Cryptosporidium*** species/genotypes across the sampled individuals**.

**Table:** 

**Diagnostic test**	***N***	***Giardia duodenalis***				***Blastocystis*** **spp**			***Cryptosporidium***		
		**% (***n***)**		**IC 95%**		**% (***n***)**		**IC 95%**	**% (***n***)**		**IC 95%**
Positives by microscopy	261	23.7 (62)		[95%CI = 18.7–29.4]		35.2 (92)		[95%CI = 29.4−41.3]	1.9 (5)		[95%CI = 0.62−4.4]
Positives by qPCR	284	64.8 (184)		[95%CI = 58.9−70.3]		88.7 (252)		[95%CI = 84.5−92.2]	1.8 (5)		[95%CI = 0.57−4.1]

**Table:** 

**Genotyping**	**Assemblages**				**Sequence Types (STs)**			**Species**			**Genotypes**
	GDH (*n* = 100)	AI	61% (61)	[50.7–70.6]	ST1	29.6% (59)	[23−36]	*C. hominis*	0.7% (2)	[0.05−0.85]	ND
		BIII	32% (32)	[23.0–42.1]	ST2	30.5% (62)				[24−37]	IaA12R9
		BIV	7% (7)	[2.9–13.9]	ST3	34.5% (70)				[28−41]	IdA20
	TPI (*n* = 47)	AI	19.1% (9)	[9.1–33.3]	ST4	2.96% (6)	[1.0−6]	*C. parvum*	0.7% (2)	[0.05−0.85]	IIcA5G3c
		AII	6.4% (3)	[1.3–17.5]	ST6	0.5% (1)				[0.01−2.2]	ND
		BIII	59.6% (28)	[44.3–73.6]	NC	2.5% (5)	[0.8−5.6]	*C. viatorum*	0.4% (1)	[0.005−0.71]	ND
		BIV	14.9% (7)	[6.2–28.3]							

*ND, Not determined*.

**Table 2:** **Associations among frequency of infection, age and sex across the individuals included in the study**.

			Sex^*^			Age (years)^*^					Community^*^ **(%)**				
			**Female (%)**	**Male (%)**	***p***	**1–4 (%)**	**5–8 (%)**	**9–12 (%)**	**≥13 (%)**	***p***	**Nuevo Paraíso**	**Puerto Nariño**	**San Juan del Soco**	**Villa Andrea**	***p***
*Giardia duodenalis* assemblages	GDH (*n* = 100)	AI	44.3	55.7	0.947	27.9	27.9	34.4	9.8	**0.033**	40	28	40.4	28.1	0.275
		BIII	43.8	56.3	0.972	56.2	18.7	15.6	9.4	0.071	15	13.4	13.5	34.4	**0.099**
		BIV	42.9	57.1	0.950	42.9	42.9	0.0	14.3	0.397	0.0	6.1	3.8	0.0	0.224
	TPI (*n* = 47)	AI	66.7	33.3	0.104	11.1	44.4	44.4	0.0	0.247	16.7	4.9	1.9	3.1	0.142
		AII	33.3	66.7	0.739	0.0	33.3	33.3	33.3	0.336	5.6	1.2	0.0	3.1	0.615
		BIII	32.1	67.9	0.080	39.3	28.6	25.0	7.1	0.626	5.6	13.4	13.5	28.1	0.135
		BIV	57.1	42.9	0.397	42.9	28.6	14.3	14.3	0.755	5.6	2.4	3.8	6.2	0.935
*Blastocystis* spp STs	(*n* = 203)^**^	ST1	37.3	62.7	0.061	42.4	27.1	20.3	10.3	**0.029**	5.9	12.8	3.9	6.4	**0.012**
		ST2	58.1	41.9	0.046	25.8	29.0	30.7	14.5	0.892	2.5	13.3	10.3	4.4	0.373
		ST3	44.9	55.1	0.594	18.6	30.0	28.6	22.9	0.056	2.5	13.8	12.3	5.9	0.142
		ST4	50.0	50.0	0.902	16.7	50.0	33.3	0.0	0.533	0.99	1.5	0.5	0.0	0.333
		ST6	100	0.0	0.292	0.0	100	0.0	0.0	0.495	0.99	0.99	0.5	0.0	0.269
*Cryposporidium Species*	(*n* = 5)	*C. hominis*	40.0	0.0	NC	40.0	0.0	0.0	0.0	0.329	0.0	40.0	0.0	0.0	NC
		*C. parvum*	40.0	0.0	NC	20.0	0.0	0.0	0.0	0.329	0.0	40.0	0.0	0.0	NC
		*C. viatorum*	20.0	0.0	NC	0.0	20.0	0.0	0.0	0.082	0.0	20.0	0.0	0.0	NC

*In bold are shown the statistical significant associations*.

* *The percentages were calculated by rows, considering the total number of individuals in each category*.

** *ST could not be identified in 2.5 % (n = 5) of the sequenced samples*.

*NC, not calculable. The estimator could not be calculated because one of the fields contained no data during the dispersion analysis*.

### Associations between sociodemographic variables and *Giardia* assemblages

The distribution of assemblages with *gdh* identified for the age groups evaluated showed a significant association and when evaluating the independent variables was found that only the assemblage AI had an association being more frequently detected in children 9–12 years old (34.4%) (Table 2). No significant associations between diagnostic methods and communities were found but when comparing the distribution of assemblages against communities was found an association, and when comparing with independent variables, the significant association was confirmed (*p* < 0.05) between the BIII assemblage and the community of Villa Andrea with 34.4% positive rate (Table 2). In the case of *tpi* marker, no association between age groups, gender and community assemblages was observed.

### Frequency of *Blastocystis* infection, STs, and alleles

**Figure 3:**
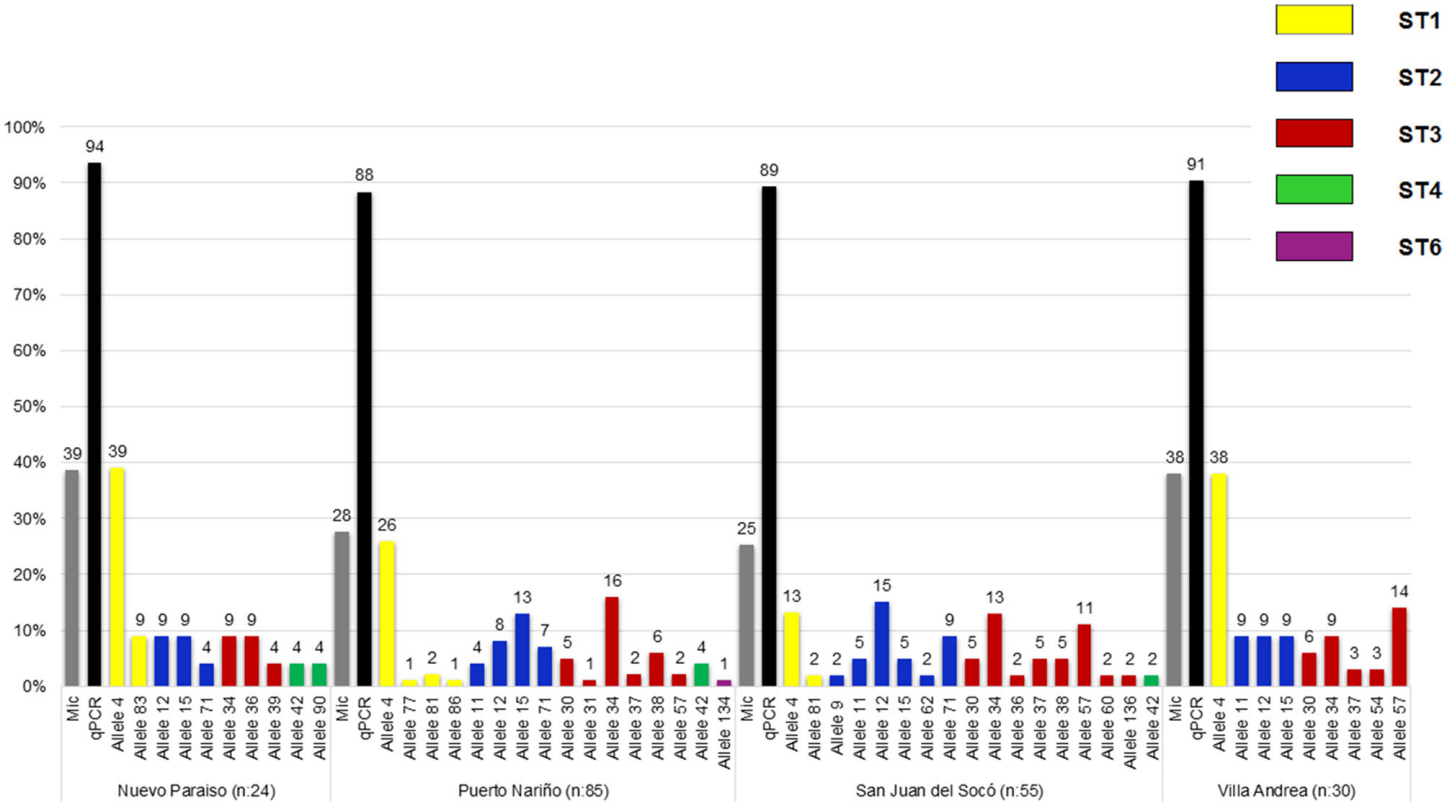
**Frequency and distribution of ***Blastocystis*** STs and alleles found by each community of the study**.

A total of 92 samples (35.2%, CI: 29.4–41.3) over 261 samples were positive for *Blastocystis* by microscopy, where the highest percentage was obtained in Puerto Nariño (40.2%) followed by Nuevo Paraíso (38.7%), Villa Andrea (38.1%) and San Juan del Socó (25.0%), for the above there were not significant associations between infection and the communities (Table 1). Real-time PCR showed 252 positive samples (88.7%, CI: 84.5–92.2) on a total of 284 samples and more frequently in the community of Nuevo Paraíso (93.5%), followed by Villa Andrea (90.7%), Puerto Nariño (87.8%) and San Juan del Socó (87.5%) (Figure 3). A total of 204 sequences were analyzed from the amplified products with the 18S rRNA and we conducted 18S barcoding observing ST3 (34.5%), ST2 (30.5%), and ST1 (29.6%) (Table 1), with the highest frequencies across all the samples. These STs were more frequently present in San Juan del Socó (35.2 and 29.6%) respectively. Additionally, ST 6 (0.9%) was found in the community of Puerto Nariño and ST4 in Nuevo Paraíso (6.9%), Puerto Nariño (2.6%) and San Juan del Socó 1.4%. Subsequently, we identified the alleles present in each ST, where allele 4 belonging to ST1 was the most common followed by allele 34 from ST3. Regarding the mixed infections, a total of 13.3% (*n* = 27) were detected observing ST1 mixed with ST2, ST3, ST4, and ST5.

### Associations between sociodemographic variables, STs, and *Blastocystis* alleles

To evaluate the distribution of STs identified for the age groups, we found that ST1 was the only one which had an association with age again being reported more frequently in the group of children under 5 years (Table 2). Subsequently, when evaluating which one(s) of alleles of this ST is the one with more association with independent variables, we found that allele 4 is having a statistically significant association between the age group under 5 years. No significant association between the variables of sex and STs and distribution of alleles was observed. In conducting the analysis between the frequencies of the STs and communities, an association where the ST1 was the most frequent with 12.8% in the community of Puerto Nariño was found and to evaluate the variables independently it was found that ST2 and ST3 have a significant association being more common in the community of San Juan del Socó. When analyzing the variables independently, no significant association was found between them for either STs.

### Frequency of *Cryptosporidium* infection, species, and subtypes

A total of 1.9% (*n* = 5) out of the 261 samples were positive for *Cryptosporidium* by microscopy. Real-time PCR showed 5 positive samples (1.8%) on a total of 284 samples (Table 1), the samples positive by microscopy were the same positive by qPCR. The 5 samples were submitted to genotyping of the Gp60 for subtypes identification. The species *C. viatorum* 0.4% (*n* = 1), *C. hominis* 0.7% (*n* = 2) (with the subtypes IdA19 and IaA12R8) and *C. parvum* 0.7% (*n* = 2) (with the subtype IIcA5G3c). All samples were detected in Puerto Nariño community. Additionally only the presence of *Cryptosporidium* in the female sex was found and no association was found with any of the sociodemographic variables. This is also attributable to the small number of positive samples obtained for this microorganism. On the other hand, when analyzing the association between positive samples for *Giardia, Blastocystis* and the other intestinal parasites detected by microscopy. We found no significant association with the presence of *Cryptosporidium* and the above mentioned parasites. Positive samples for *C. hominis* and *C. parvum* were found in the age groups of children under 5 years old and *C. viatorum* in children aged between 5 and 8 years (Table 2).

### Genetic diversity indexes in *Giardia* and *Blastocystis*

We calculated the genetic diversity indexes per community across the *gdh* and *tpi* markers for *Giardia* and 18S rRNA marker for *Blastocystis* (Table 3). Tests of significance between the results of community diversity indices (as independent samples), helped to identify a lack of statistically significant differences between the results of π by community. In the case of θ and Hd profiles of differential association between the results obtained by the markers used for each species were found. The results of the index θ using the results of the marker gdh (*G. intestinalis*), obtained for the San Juan del Soco community, showed a significant difference to the results of Nuevo Paraiso and Villa Andrea. With the same score (*gdh*) a significant difference between the results of San Juan del Soco and Puerto Nariño were found. The results of the second marker used for typing *G. intestinal* (*tpi*), showed a significant difference between the results obtained between San Juan del Soco, Puerto Nariño and Nuevo Paraiso. Significant differences were found with Hd for *tpi* between Nuevo Paraiso and the remaining three populations, but also between San Juan del Soco and Puerto Nariño. In Addition, Nuevo Paraiso showed significant differences with the results of the other three communities for both θ and Hd, calculated for the sequences obtained for 18S (*Blastocystis*) and additional significant difference was identified for Hd calculated for San Juan del Soco and Puerto Nariño, from the results of 18S (Table 4). Despite these findings, no significant differences between the variances of rates among these populations were identified, so the hypothesis of equality of the results of the indices of genetic diversity at the population level is not rejected among these communities.

**Table 3:** **Genetic diversity indexes of ***tpi*** and ***gdh*** for ***Giardia*** and ***18S*** rDNA for ***Blastocystis*** across the communities sampled in the study**.

**Community**	***Giardia***												***Blastocystis***					
	***gdh***						***tpi***						***18S***					
	***n***	**π**	**θ**	**SD-θ**	**Hd**	**SD-Hd**	***n***	**π**	**θ**	**SD-θ**	**Hd**	**SD-Hd**	***n***	**π**	**θ**	**SD- θ**	**Hd**	**SD-Hd**
Nuevo Paraíso	11	0.05082	0.04594	0.01907	0.945	0.054	6	0.18463	0.16612	0.07856	1.000	0.096	24	0.15537	0.20393	0.06647	0.996	0.013
Puerto Nariño	40	0.06885	0.10569	0.03142	0.894	0.044	18	0.10855	0.11842	0.04153	0.993	0.021	85	0.0878	0.13804	0.03499	0.972	0.008
San Juan del Soco	32	0.0740	0.12605	0.03904	0.839	0.065	10	0.05642	0.08532	0.03506	1.000	0.045	55	0.09551	0.15227	0.04177	0.988	0.006
Villa Andrea	20	0.06699	0.06034	0.02134	0.911	0.054	14	0.06025	0.10911	0.0408	0.989	0.031	34	0.09597	0.1152	0.03489	0.998	0.008
Total	103	0.06842	0.11628	0.02887	0.888	0.029	48	0.09736	0.12286	0.03471	0.998	0.005	198	0.08328	0.15432	0.0343	0.921	0.011

*n, Number of sequences*.

*Hd, Haplotype diversity*.

*SD, Standard Deviation*.

**Table 4:** **Statistical analyses of genetic diversity indexes across the communities studied**.

			π				θ				**Hd**			
		**Community**	**Nuevo Paraíso**	**Puerto Nariño**	**San Juan del Soco**	**Villa Andrea**	**Nuevo Paraíso**	**Puerto Nariño**	**San Juan del Soco**	**Villa Andrea**	**Nuevo Paraíso**	**Puerto Nariño**	**San Juan del Soco**	**Villa Andrea**
*Giardia*	*gdh*	Nuevo Paraíso		1.0794	1.0705	1.0460		0.0939	**0.0208**	0.736		0.3481	0.5489	1.0460
		Puerto Nariño			1.0104	0.9635			0.2153	0.730			**0.0217**	0.2745
		San Juan del Soco				0.9735				**0.0064**				0.3996
		Villa Andrea												
	*tpi*	Nuevo Paraíso		1.1055	1.0610	1.0892		**0.0431**	**0.0358**	0.0527		**0.0000**	**0.0479**	**0.0010**
		Puerto Nariño			1.0482	0.9814			0.6183	0.9653			**0.0068**	0.1335
		San Juan del Soco				1.0300				0.6596				0.2154
		Villa Andrea												
*Blastocystis*	*18S*	Nuevo Paraíso		1.0506	1.0378	1.0183		**0.0000**	**0.0052**	**0.008**		**0.0013**	**0.0000**	**0.0107**
		Puerto Nariño			1.0143	0.9662			0.1433	1.0181			**0.0248**	0.9662
		San Juan del Soco				0.9799				0.2709				0.0593
		Villa Andrea												

*Bold values indicate statistically significant*.

### Mixed infections in *Giardia* and *Blastocystis*

**Figure 4:**
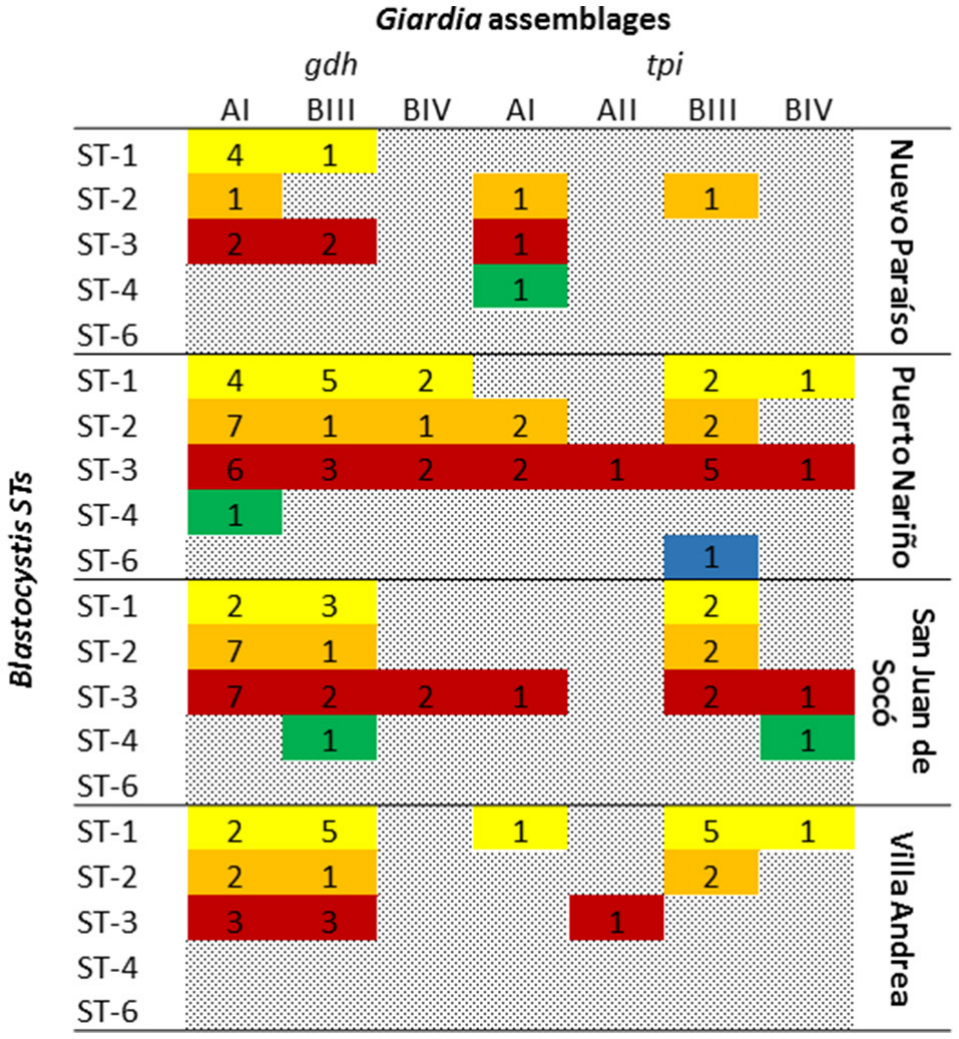
**Mixed infections between ***Blastocystis*** subtypes and ***G. intestinalis*** assemblages**.

Mixed infections between *G. intestinalis* and *Blastocystis* were found by microscopy in 19.6% (*n* = 29) being more common in Puerto Nariño with 27% (*n* = 20), followed by Villa Andrea 16% (*n* = 4), Nuevo Paraíso 14.3% (*n* = 2) and San Juan del Socó 8.6% (*n* = 3). At the time of the analysis against socio-demographic factors, community, sex and age were not statistically significant. In addition to the analysis for a possible association between subtypes of *Blastocystis* and *G. intestinalis* assemblages, did not show any association. The most frequent co-infections among STs of *Blastocystis* and *G. intestinalis* assemblages were evident; the STs 1, 2, and 3 present infection with assemblage AI, the ST3 was present in all assemblages, in the case of ST4 with AI, BIII, and BIV assemblages. Finally ST6 with a single report in Puerto Nariño presented BIII assemblage infection. A higher frequency of mixed infections in the community of Puerto Nariño and San Juan del Soco was observed (Figure 4).

## Discussion

The diagnosis of gastrointestinal parasites has traditionally been based on feces microscopy and the use of conventional techniques based on concentration for the subsequent visualization of parasitic stages. This leads to obtain a variability in sensitivity and diagnostic specificity, being totally dependent of the observer's expertise (Mejia et al., 2013). The object then is to improve and/or strengthen the diagnosis of parasitic infections, for which the use of molecular tools arises due to its high sensitivity allowing specific identification of various pathogens, i.e., species identification and subpopulations as is the case of *Giardia* assemblages, *Blastocystis* STs and *Cryptosporidium* species. This translates into specific and timely treatment to the organism and better clinical management of the patients. One of the applied/used strategies by different research groups is the molecular epidemiology. It has proven to be of great importance as it helps to understand the transmission dynamics and genetic determinants of parasitic infectious diseases (Ramírez et al., 2012). For example, recent reports have shown that giardiasis in childhood is associated with malnutrition, stunting and impaired cognitive development, regardless of the presence of diarrhea, prevalent among children two to five years old, according to several reports from developing countries (Montresor, 2002). It is clear that the incidence of intestinal parasites is higher in children than in adults because of the lack of natural resistance and differences in their behavior and habits (World Health Organization, 2012). The above closely related to the environmental determinants and socio-economic characteristics of a population, which generate a higher risk of infection. The World Health Organization (WHO) in its action plan raises early protection of children and vulnerable communities; detection, treatment and control of intestinal parasites are subject to follow, because this is one of the leading causes of morbidity and mortality in children worldwide (Haque et al., 2005; Ministerio de Salud y Protección Social, 2015).

An immune response, which can be individually variable and influenced by nutritional status, genetic factors, and repeated exposure to *G. intestinalis* suggesting that, contributes to low detection rates of *Giardia* seen in older children as well as an explanation to the asymptomatic infections (Forsell et al., 2016). Our results are in accordance with these premises, where we observed high rates of infection for *Giardia* (23.7% by microscopy and 64.8% by qPCR) and *Blastocystis* (35.2% by microscopy and 88.7% by qPCR) and also for other parasites such as *Entamoeba, Ascaris, Trichuris*, and hookworms. Also, the frequencies of infection for *Giardia* and *Blastocystis* are in accordance to other reports in Colombia showing that in the Amazon region *Giardia* has a frequency of 37.3% and *Blastocystis* has a frequency of 40.7%, comparable to the frequencies of the country being for *Giardia* 15.4% and *Blastocystis* 57.7% (Ministerio de Salud y Protección Social, 2015). The differences are striking in terms of the sensitivity of the techniques which are also supporting the fact of using molecular methods instead of conventional microscopy as has been clearly stated by different authors (Bertrand et al., 2005; Asher et al., 2012; Mejia et al., 2013; Boadi et al., 2014; Ramírez et al., 2014). These can be explained by the intermittent excretion of cysts in *Giardia* and also the expertise of the operator who implements microscopy.

The geographical distribution of the *G. intestinalis* assemblages is intriguing since there is a lack of geographical structuring across the globe (in the case of the human-infective assemblages A and B). In central and south-America, there exist areas with differential distribution of assemblages. Some reports from Mexico, Brazil and Colombia identifies higher frequencies of assemblage A (Eligio-Garcia et al., 2008; Kohli et al., 2008) while in Nicaragua, Argentina and also Colombia some authors report the predominance of assemblage B (Lebbad et al., 2008; Minvielle et al., 2008; Arroyo-Salgado et al., 2014; Ramírez et al., 2015). This suggests that the distribution is not geographically associated and is more linked to the socioeco-epidemiological factors of the population studied. This is corroborated by our findings where the most frequent assemblage was AI (61%), contrasting the different findings in three sympatric regions of the country. For example in the Caribbean and central region the most frequent assemblage was B (Arroyo-Salgado et al., 2014; Ramírez et al., 2015) but in the Andean region assemblage A was more frequent (Rodríguez et al., 2014). Intriguingly, our results showed that the most frequent sub-assemblage was AI (61%) that has been more associated to animals than humans. This could be attributed to the lack of water treatment of the population studied and a close contact with wild animals as is frequent in the amazon region. The presence of AI could be also possible due to contamination of public water with raw sewage from animal and human sources (Volotão et al., 2007; Helmy et al., 2009). It is clearly that the indigenous populations studied lack the access to potable water that is highlighted in our findings.

Our results also showed that after AI, assemblage BIII was the second most frequent. Curiously, this assemblage is highly associated to infections in dogs and cats which might be suggesting a strong zoonotic transmission in the community of Villa Andrea with 35.5% positivity in children under 5 years (Table 1; Lebbad et al., 2008; Ryan and Caccio, 2013). Regarding the genetic diversity indexes is observed that San Juan del Socó is the community with the highest genetic diversity and the second community with the highest rate of *Giardia* infection (65%), and assemblage AI was the most frequent (40.4%). Curiously, this community is the farthest from the municipality of Puerto Nariño suggesting that active enzootic transmission might be occurring representing a hot-spot of diversity where distinct genotypes from animals might be transmitted to humans.

The existence of many subtypes within families of *C. hominis* and *C. parvum* supports the complexity of *Cryptosporidium* transmission and its zoonotic potential in developing countries (Leav et al., 2002; Gatei et al., 2007). The subgenotypes considered anthroponotic (IIc and IIe) are the most common, along with the IId zoonotic subgenotype and the subgenotype IIc present in human infections (Akiyoshi et al., 2006; Sharma et al., 2013). These data supports our findings of IdA19 and IaA12R8 in *C. hominis* and IIcA5G3c in *C. parvum*. Herein, we show the first report of *C. viatorum* in Colombia; the data collected in different studies indicate the presence of *C. viatorum* in Asia (Nepal, Bangladesh, India, Pakistan, and potentially Dubai), Africa (Nigeria, Ethiopia and Kenya) and Central America and the Caribbean (Guatemala and Barbados) (Stensvold et al., 2015). *C. viatorum* is currently the only species of *Cryptosporidium* that has been reported only in humans but the data are still insufficient to ensure that this species is restricted to human infections; also presumed presents a global distribution (Elwin et al., 2012). Previous studies indicate that the *C. viatorum* species has a genetic variation which may be related to differences in the geographical origin of the infection in different regions where it has been found (Elwin et al., 2012).

On the other hand, a high frequency of *Blastocystis* was observed in our study (88.7%; Table 1), which agrees with previous reports of infection by *Blastocystis* in Colombia (Arias et al., 2010; Ramírez et al., 2014). In addition, this frequency is very similar to other countries in South America ranging from 40 to 70% in humans (Jiménez et al., 2012; Ramírez et al., 2016). Regarding the distribution of STs in humans, a study in South America showed a vast diversity occurrence of STs (1, 2, 3, 4, 5, 6, 7, 8, and 12). In the case of Colombia have been reported STs 1, 2, 3, 4, 6, and 7 in humans (Malheiros et al., 2011; Ramírez et al., 2016). Our results show the predominance of STs 1, 2, and 3 which is consistent with previous reports even in indigenous communities such as Mato Grosso, Brazil (Malheiros et al., 2011). Herein, we show several important findings which are the description of ST4 in indigenous communities since this ST is more common in Europe and less frequent in South America. In addition, ST6 that is more common in birds and may be an evidence of zoonotic transmission across the population studied (Stensvold et al., 2007). When the results of the 18S alleles were retrieved, we observed that allele 4 from ST1 was frequent across the four communities; this allele has already been described in Colombia and with a frequency of 9% in all South America. Also, this allele has been reported in *Didelphis marsupialis* from Colombia supporting again a plausible zoonotic transmission (Ramírez et al., 2014, 2016). In the case of the ST2, alleles 12 and 15 were observed in all the communities as has already been described in South America with frequencies of 14 and 4% respectively, but intriguingly San Juan del Socó showed a higher number of ST2 alleles (6 in total) and in accordance with the genetic diversity indexes already described (Table 3; Figure 3). Also, allele 9 was found in this community and previously described in dogs and rats suggesting again a possible zoonotic profile (Ramírez et al., 2014). Regarding the ST3, the most frequent allele was 34, which has been reported in Colombia and with a frequency of 12% in South America (Ramírez et al., 2016). Interestingly, this allele has been associated to urticaria in Argentinean patients and also to cattle in Colombia (Ramírez et al., 2014; Casero et al., 2015). For ST4, we found alleles 42 and 90, the allele 42 has been described worldwide but allele 90; is novel in our country because dogs and cats have been found infected with alleles 42 and 133 (Alfellani et al., 2013; Stensvold and Clark, 2016). Finally, for ST6 we identified allele 134 in Puerto Nariño which is a novel description for the country because there is only a report of allele 122 (Ramírez et al., 2016).

According to the results obtained with mixed infections between *Blastocystis* and *G. intestinalis* (Figure 4) once again puts in evidence the need to employ techniques based on the use of different molecular markers to conduct a real identification of different subpopulations present in a specific area, and achieve associations regarding risk factors (Ryan and Caccio, 2013). Additionally, in the case of mixed infections, it is highlighted the frequency of infection with ST5, its presence in Latin America is only described in Bolivia 20% (*n* = 8; Ramírez et al., 2016), previously it had been reported in the UK, Australia and Pakistan (Yakoob et al., 2010; Wang et al., 2014). The importance of this ST is the zoonotic potential, as had previously been described in infections in pigs and cattle (Yan et al., 2007), reaffirming the importance in direct relation to human activities and possible infections that can occur between different epidemiological scenarios. In short, it is worth noting the fact of finding a high rate of mixed infections 13.7%, this being an indicator of active transmission by generating new MLGs as observed in *Trypanosoma cruzi* (Ramírez et al., 2012).

The Amazon basin is the largest in the world and is the fifth freshwater reserve on the planet. Contradictorily, people living in these areas have poor quality of life, which favors the infection of diseases of fecal-oral transmission. Therefore, proper control of giardiasis, blastocystosis and cryptosporidiosis particularly in the Amazon region requires improving the quality of drinking water and reducing environmental contamination by feces, and control of the close contact with wildlife; despite of its high prevalence and its impact on health worldwide, enteric protozoa infections emerge as neglected because conditions are not taken into account as the controls used for infections caused by helminths. In different studies have shown that people living near a water body like a river, are at greater risk of becoming infected with some intestinal parasite as in the case of *G. intestinalis* and *Blastocystis*. In a recent study from the Brazilian amazon, the authors found a predominance of assemblage A which is in accordance with our findings (Coronato Nunes et al., 2016). This highlights the importance of our findings, and shows the need to implement strategies for control and prevention in the communities studied because they are considered to be vulnerable indigenous communities.

In conclusion, we employed molecular methods and high-resolution genotyping to unravel the transmission dynamics of *Giardia, Blastocystis*, and *Cryptosporidium* across four indigenous communities of the Colombian Amazon basin. We observed a high profile of zoonotic transmission regarding the *Giardia* assemblages and *Blastocystis* STs/alleles. Also, we highlight the elevated frequency of infection by these two protozoans suggesting an active transmission in the area. San Juan del Socó was the community with the highest genetic diversity for both pathogens and the closest to the jungle space. Our findings reinforces the need to deploy better epidemiological surveillance systems for enteric pathogens in the area and the use of molecular epidemiology for a vast comprehension of the transmission dynamics including the zoonotic potential of these species.

## Author contributions

JR analyzed the data, revised the final version and supervised the project. NG, JT, LS, ASal, CR, and MR analyzed the samples by microscopy. ASan wrote the manuscript, analyzed the data and conducted the molecular biology experiments. MM conducted the statistical analyses. PR and ML revised the manuscript and analyzed the data. LX and YQ conducted the typing of Cryptosporidium.

## Funding

We thank Dirección de Investigación Universidad Nacional de Colombia grant number 22946 for funding this project.

### Conflict of interest statement

The authors declare that the research was conducted in the absence of any commercial or financial relationships that could be construed as a potential conflict of interest.
